# Rapidly progressive adult-onset neuronal intranuclear inclusion disease beginning with autonomic symptoms: a case report

**DOI:** 10.3389/fneur.2023.1190981

**Published:** 2023-05-25

**Authors:** Yi Zhu, Qian Yang, Yun Tian, Weibing Fan, Xinfa Mao

**Affiliations:** ^1^Department of Anesthesiology, Hunan Children's Hospital, Changsha, Hunan, China; ^2^Department of Neurology, The Third Hospital of Changsha, Changsha, Hunan, China; ^3^Department of Neurology, Xiangya Hospital, Central South University, Changsha, Hunan, China

**Keywords:** neuronal intranuclear inclusion disease, autonomic symptom, pathological change, case report, misdiagnose

## Abstract

**Background:**

Neuronal intranuclear inclusion disease (NIID) is a rare neurodegenerative disease that can affect the nervous and other systems of the body. Its clinical manifestations are complex and easily misdiagnosed. Adult-onset NIID beginning with autonomic symptoms such as recurrent hypotension, profuse sweating, and syncope has not been reported.

**Case presentation:**

An 81-year-old male was admitted to the hospital in June 2018 due to repeated episodes of hypotension, profuse sweating, pale complexion, and syncope for 3 years, and progressive dementia for 2 years. DWI was not possible due to the presence of metal residues in the body. Cutaneous histopathology revealed sweat gland cell nuclear inclusions and immunohistochemistry showed p62 nuclear immunoreactivity. Blood RP-PCR identified an abnormal GGC repeat expansion in the 5′UTR of the *NOTCH2NLC* gene. Accordingly, this case was diagnosed as adult-onset NIID in August 2018. The patient subsequently received vitamin C nutritional support, rehydration, and other vital signs maintenance treatments during hospitalization, but the above symptoms still recurred after discharge. With the development of the disease, lower extremity weakness, slow movement, dementia, repeated constipation, and vomiting appeared successively. In April 2019, he was hospitalized again for severe pneumonia, and died of multiple organ failure in June 2019.

**Conclusion:**

The presented case exemplifies great clinical heterogeneity of NIID. Some patients may have neurological symptoms and other systemic symptoms simultaneously. This patient started with autonomic symptoms, including recurrent episodes of hypotension, profuse sweating, pallor, and syncope, which progressed rapidly. This case report provides new information for the diagnosis of NIID.

## Introduction

Neuronal intranuclear inclusion disease (NIID) is a rare chronic neurodegenerative disease that affects the central and peripheral nervous systems, as well as other organs throughout the body. Histopathological features of NIID include intranuclear inclusions found in the affected organs and tissues such as skin, internal organs, and skeletal muscle. Diffusion-weighted imaging (DWI) can reveal hyperintense lesions at the corticomedullary junction area. Further, genetic testing can reveal GGC trinucleotide repeat expansion in the *NOTCH2NLC* gene. We present an atypical case of adult-onset NIID. The patient began to suffer from repeated hypotension, pallor, profuse sweating, and syncope, and was misdiagnosed many times. Finally, we made a diagnosis of NIID through skin biopsy and genetic testing. This report of a rare case of NIID with onset of autonomic symptoms will help to better understand the clinical features of this disease and make an early and accurate diagnosis.

## Case presentation

An 81-year-old male was admitted to our hospital in June 2018 due to recurrent episodes of hypotension, profuse sweating, pale complexion, and syncope for 3 years and progressive dementia for 2 years. These symptoms beginning 2015 were not related to body posture. Blood pressure lower than 90/60 mmHg occurred several times during the episode, even undetectable in severe cases. Consciousness typically returned within 10 min. Pallor, profuse sweating, and hypotension usually fully recovered within 30 min. Before the episode, the patient had no limb movement disorder, dizziness, diplopia, slurred speech, palpitations, and chest pain. During the episode, the patient had no limb twitching, foaming at the mouth, and urinary and fecal incontinence, and multiple blood glucose test results were 6–16 mmol/L. About 1 year after the onset of autonomic symptoms, cognitive decline, weakness of limbs, unsteady walking, repeated vomiting, and intractable constipation gradually appeared.

**Figure 1:**
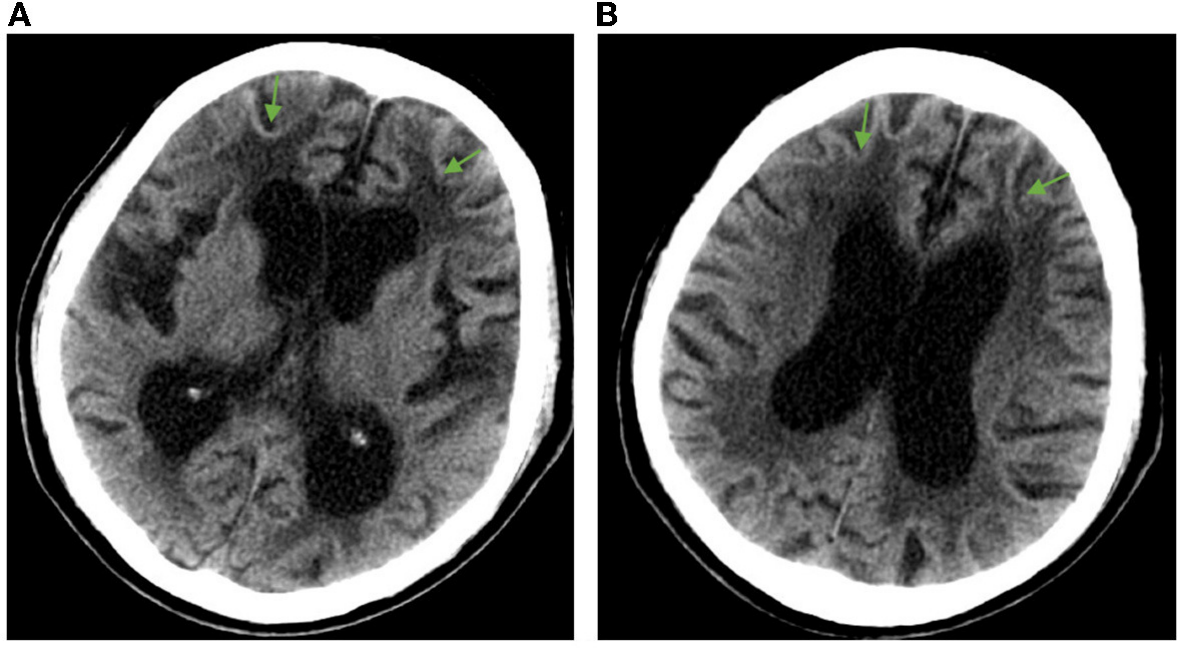
Head CT imaging showing brain atrophy and white matter lesions in the patient. **(A)** Enlargement of the anterior and posterior horns of the lateral ventricles and widening of the sulcus, with arrows showing white matter lesions around the anterior horns of the lateral ventricles. **(B)** Enlarged body of the lateral ventricle with arrows showing white matter lesions around the body of the lateral ventricle.

**Figure 2:**
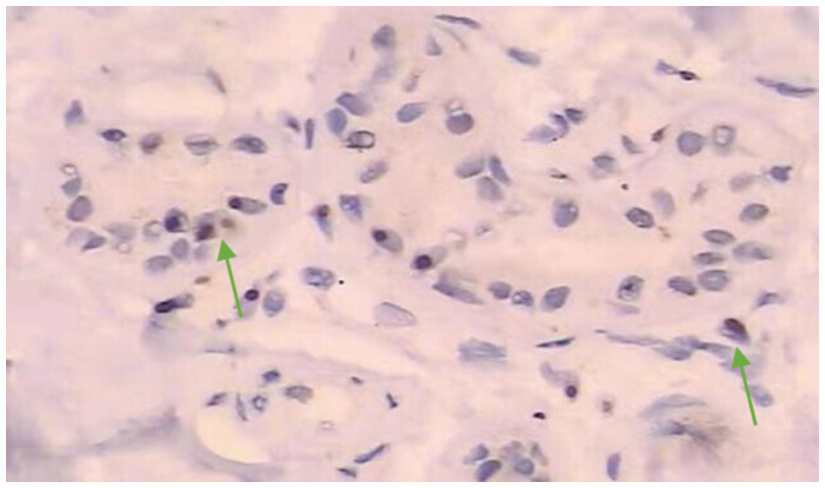
Histopathological examination of skin biopsy after admission showed round or nearly round inclusion bodies in the nucleus of sweat gland cells (magnification, ×200). Immunostaining displayed intranuclear inclusions with p62 (mouse antibody, 610833, BD Biosciences) immunoreactivity.

**Figure 3:**
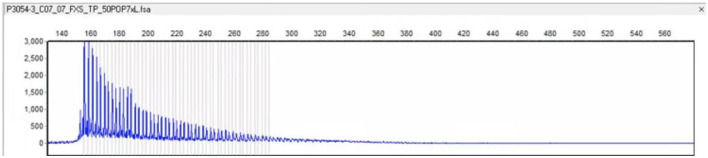
Genetic testing by RP-PCR revealed an aberrant GGC repeat expansion in the 5′UTR of the *NOTCH2NLC* gene.

Admission physical examination: blood pressure 120/80 mmHg, heart rate 80 beats/min, no obvious abnormalities in heart, lung, and abdomen. Neurological exam: decreased recent and remote memory, acalculia, and decreased spatiotemporal orientation. According to the Medical Research Council (MRC) scale, the patient's muscle strength was grade 5 in both upper limbs and grade 4 in both lower limbs. Muscle tone was normal in the four limbs, but the deep reflexes of the two lower limbs were symmetrically weakened. Negative pathological signs, positive Romberg's sign, and normal finger-nose and heel-knee-shin tests. Walking with the aid of a walker, the patient exhibited a wide-based gait. Mini-Mental State Examination score was 7. Head computed tomography (CT) scan on admission showed brain atrophy and white matter lesions (Figures 1A, B). Dynamic electrocardiogram revealed sinus rhythm, occasional atrial premature beats, incomplete paired beats, transient atrial tachycardia, and occasional ventricular premature beats. Echocardiography revealed thickened ventricular septum, decreased aortic elasticity, and decreased left ventricular diastolic function with an ejection fraction of 75%. Routine blood test: red blood cell count 3.53 × 10^12^/L, hemoglobin 110 g/L, hematocrit 32.3%, albumin 30.40 g/L, low-density lipoprotein cholesterol 1.87 mmol/L, random blood glucose 12.48 mmol/L, and estimated glomerular filtration rate 71.12 mL/min/1.73 m^2^. Cardiac enzymes, troponin, brain natriuretic peptide, electrolytes, coagulation tests, thyroid function, and β-hydroxybutyric acid were normal. Urinalysis revealed leukocyte count 178.90 μL. Stool routine examination was normal. DWI was not performed due to the presence of metal residues in the body. Skin biopsy indicated intranuclear inclusion bodies in eccrine sweat gland cells, fibroblasts, and adipocytes. p62 immunohistochemistry demonstrated nuclear immunoreactivity (Figure 2). Finally, genetic testing by repeat-primed polymerase chain reaction (RP-PCR) found that the patient had GGC repeats in the 5′UTR of the *NOTCH2NLC* gene (Figure 3). The patient was therefore diagnosed with adult-onset NIID.

During hospitalization, the patient was mainly given supportive care to maintain vital signs. Due to long-term malnutrition, the treatment plan included supplementing vitamin C and dextrose and sodium chloride injection, increasing oxygen supply though oxygen inhalation, and maintaining normal blood pressure and electrolyte balance. Since the onset of the disease, the patient manifested progressive aggravation of symptoms such as dementia and limb weakness, repeated vomiting, and intractable constipation, accompanied by recurrent episodes of autonomic symptoms such as pale complexion, excessive sweating, hypotension, and syncope.

For some symptoms such as nausea and vomiting, the patient received symptomatic treatment. In 2019, the patient was already severely demented and bedridden. He was readmitted to our hospital in April with severe pneumonia. Eventually, he developed multi-organ failure and died in June 2019 at the age of 82.

## Discussion

NIID is a rare slowly progressive neurodegenerative disease first described by Lindenberg and colleagues in 1968 (1). NIID is highly clinically heterogeneous and its pathological characteristics include the presence of eosinophilic hyaline intranuclear inclusions in the central and peripheral nervous systems and internal organs. Eosinophilic p62/ubiquitin-positive intranuclear inclusions in neurons and other somatic cells can be observed under light microscope, and dense membraneless filamentous substances can be seen under electron microscopy (2). Moreover, high signal intensity in the corticomedullary junction on DWI is characteristic of NIID (3). According to the age of onset, NIID can be classified into infantile, juvenile, and adult forms. Infantile and juvenile patients often present with ataxia or aberrant mental behavior as the first manifestation (4–7), while adult-onset patients usually begin with dementia or limb weakness as initial clinical symptoms. The adult form can be further divided into sporadic and familial subtypes. Sone *et al*., summarized 38 sporadic adult-onset NIID cases, aged 51–76 years, with dementia onset as the main symptoms (94.7%), followed by limb tremor, ataxia, abnormal behavior, disturbance of consciousness, and encephalitis-like seizures (8, 9). Others reported NIID with Parkinson-like symptoms (10), abnormal emotional behavior (11), chronic headache (12), sensory disturbance (13), epileptic episodes (14), cardiomyopathy, constipation and gastroenteritis, bronchopneumonia and respiratory failure (15), and dysphagia (12). However, there were no reports of early autonomic symptoms such as hypotension, pallor, profuse sweating, and syncope in patients with NIID.

The patient we reported here initially presented with hypotension (< 90/60 mmHg) and autonomic symptoms unrelated to postural changes. At the early stage of the onset, dynamic electrocardiograms showed no malignant arrhythmias such as cardiac arrest and conduction block, and cardiogenic syncope was ruled out. At the time, vasovagal syncope was considered. The patient had metal implants in his body that prevented an MRI of the head. At the same time, the patient's early symptoms were atypical, which brought great difficulties to clinical diagnosis. As the condition worsened, the patient subsequently developed dementia, weakness in both lower limbs, slow movement, repeated vomiting, and intractable constipation. The patient' symptoms and physical examination results suggested that the lesion may involve multiple systems and sites, such as autonomic nerves, peripheral nerves, cerebellum or extrapyramidal system, and cerebral cortex. After reviewing the literature, the patient was considered likely to have NIID. Subsequent dermatopathology and immunohistochemistry revealed p62-positive intranuclear inclusions in eccrine sweat gland cells, fibroblasts, and adipocytes (Figure 2) and genetic testing detected GGC repeat expansion (Figure 3), both confirming adult NIID diagnosis. Therefore, all symptoms of the patient can be reasonably explained.

NIID is genetically and phenotypically heterogeneous and can affect the nervous, circulatory, urinary, and digestive systems. In some patients, other systemic symptoms preceded neurologic symptoms, suggesting systemic heterogeneity in NIID (16). Current studies believe that NIID has multiple phenotypes, such as dementia, tremor, Parkinson-like, amyotrophic lateral sclerosis-like, oculopharyngodistal myopathy-like, and Charcot-Marie-Tooth-like phenotypes (17), which increase the difficulty of diagnosis. In most patients with NIID, a hyperintense foci at the gray-white matter junction on DWI is an important diagnostic marker of NIID since it does not disappear throughout the course of the disease (8). Detection of ubiquitin-positive eosinophilic intranuclear inclusions in skin biopsies and GGC repeat expansion in the *NOTCH2NLC* gene can further aid in accurate diagnosis. At present, there are no proven treatments for NIID. Most NIID progress slowly. However, this patient progressed rapidly, became bedridden, and died five years later of severe pneumonia and multi-organ failure. The rare onset and rapid progression of the disease in this patient warrants further study.

## Data availability statement

The original contributions presented in the study are included in the article/supplementary material, further inquiries can be directed to the corresponding authors.

## Ethics statement

The studies involving human participants were reviewed and approved by Ethics Committee of Changsha Third Hospital. The patients/participants provided their written informed consent to participate in this study. Written informed consent was obtained from the individual(s) for the publication of any potentially identifiable images or data included in this article.

## Author contributions

YZ and QY wrote the report. WF performed diagnostic testing. XM assisted in diagnosis. QY collected data and made diagnosis. YT confirmed pathological diagnosis. All authors contributed to the article and approved the submitted version.
